# The Impact of *Bacillus subtilis* PB6 and Chromium Propionate on the Performance, Egg Quality and Nutrient Metabolizability of Layer Breeders

**DOI:** 10.3390/ani11113084

**Published:** 2021-10-28

**Authors:** Otoniel Souza, Carine Adams, Beatriz Rodrigues, Alexandre Krause, Renata Bonamigo, Kelen Zavarize, Catarina Stefanello

**Affiliations:** 1 Department of Animal Science, Federal University of Santa Maria, Santa Maria 97105-900, Brazil; otonielflx@hotmail.com (O.S.); carineadamsb@gmail.com (C.A.); bizinha75@gmail.com (B.R.); 2 Clinical Analysis Laboratory (LACVET), Federal University of Santa Maria, Santa Maria 97105-900, Brazil; alexandrekrause@ufsm.br (A.K.); bonamigo.vet@gmail.com (R.B.); 3 Animal Nutrition and Health South America, Kemin Industries Inc., Valinhos 13279-450, Brazil; Kelen.Zavarize@kemin.com

**Keywords:** blood biochemistry, breeder hen, eggshell quality, organic chromium, probiotic

## Abstract

**Simple Summary:**

Environmental conditions can generate heat stress in poultry production. Feed additives have been supplemented in diets for layers and broilers to improve their health status, immune system and nutrition, as well as productive performance. This paper shows the effects of probiotic *Bacillus subtilis* PB6 and chromium propionate diet supplementation on productive parameters, egg and eggshell quality, excreta moisture, cortisol and serum biochemistry of laying breeder hens. Diets supplemented with *Bacillus subtilis* PB6, chromium propionate or a combination of the two resulted in improved egg production, feed conversion ratio, eggshell quality and nutrient metabolizability, without modifying the main serum biochemical parameters of hens from 55 to 70 weeks of age. Highlighted improvements in hen performance and eggshell quality were observed when hens were fed diets supplemented with a combination of the probiotic and chromium. This study expands our understanding concerning the combined supplementation of probiotics and organic chromium for poultry.

**Abstract:**

The objective of this study was to evaluate the effects of *Bacillus subtillis* PB6, chromium propionate or a combination of the two on the performance, egg and eggshell quality, nutrient metabolizability and serum biochemistry of layer breeders. White Plymouth Rock and Red Rhodes Island breeder hens at 55 weeks of age were allocated in individual cages using a completely randomized block design with 16 replicates. Hens were fed control, control + probiotic (500 g/ton of *Bacillus subtilis* PB6), control + CrProp (50 g/ton of chromium propionate) and control + probiotic + CrProp diets from 55 to 70 weeks of age. Productive parameters and eggshell quality as well as cortisol and blood biochemistry were grouped each 28 d as well as for the overall period. The metabolizability of nutrients and energy was determined at 70 weeks of age. In the overall period, hens fed the control + probiotic or control + probiotic + CrProp diets had significantly higher egg production, egg mass, shell percentage, thickness and shell strength. The metabolizability of dry matter, nitrogen and energy increased in hens that were fed the control + probiotic + CrProp diet. In conclusion, diets supplemented with *Bacillus subtillis* PB6 and chromium propionate resulted in improved productive performance, eggshell quality and nutrient metabolizability of layer breeders, without modifying serum cortisol, albumin and triglycerides.

## 1. Introduction

Feed additives have been increasingly used in animal nutrition due to their remarkable benefits. The use of probiotics has aroused widespread interest in poultry farms, being related to improvements in the immune system of hens, while beneficially affecting the hosts by modifying their intestinal microbiome as well as improving feed efficiency, digestion and production performance [1,2,3].

Probiotic effectiveness for poultry may depend on several factors, including microbial species composition, diets, supplemental dose, combination with other additives and environmental stress factors [4]. *Bacillus subtilis* PB6 is a natural strain isolated from healthy chicken gut that produces antimicrobial components with broad activity against microorganisms [5,6]. In laying hens, *Bacillus subtilis* PB6 increased feed efficiency and reduced excreta moisture as well as cholesterol content in egg yolk and serum [7,8]. Other beneficial effects of this probiotic for hens were improvements in egg production, egg quality, nutrient digestibility and return on investment [9,10,11].

Heat stress is considered as one of the most important environmental stressors that modifies the oxidant/antioxidant system and compromises the health status of laying hens, resulting in their poor performance worldwide [12]. It has been reported that dietary *Bacillus subtilis* supplementation could reduce heat stress in birds [13]. However, organic chromium has been the main additive that has demonstrated positive effects against stress conditions in birds; several researchers have started exploring the effect of chromium on laying hens, breeders and broilers, for different requirements [14,15,16]. The US Food and Drug Administration approved chromium propionate (CrProp) as an additive for broiler feeds at concentrations up to 0.20 mg Cr/kg [17]. 

Chromium has been supplemented from different sources and levels, and it has been shown to present antioxidant properties, which help to attenuate the negative effects of oxidative stress, while contributing to lipid, protein and nucleic acid metabolism [18]. Furthermore, chromium has also been related to improvements in cell preservation and immune responses, which contribute to animal homeostasis and thermoregulatory capacity under heat stress conditions [19]. In laying hens, chromium propionate supplementation resulted in increased egg production, feed efficiency and egg quality, whereas it reduced egg cholesterol levels and serum cortisol [16,20,21].

The effect of *Bacillus subtilis* or chromium propionate on performance improvements in broilers is well-documented; however, more data are needed on when these additives are supplemented in diets for layer or breeder hens as well as when both additives are combined. The objective of this study was to evaluate the effects of *Bacillus subtillis* PB6, chromium propionate or a combination of the two on the performance, egg and eggshell quality, nutrient metabolizability and serum biochemistry of laying breeder hens of 55 to 70 weeks of age.

## 2. Materials and Methods

All procedures involving live-bird management and healthcare were approved by the Ethics and Research Committee of the Federal University of Santa Maria, Santa Maria, Rio Grande do Sul, Brazil (approval number: 5404280717).

### 2.1. Birds and Treatments

A total of 32 White Plymouth Rock and 32 Red Rhodes Island laying breeder hens at 50 weeks of age, obtained from the Poultry Science Laboratory (LAVIC-UFSM, Santa Maria, RS, Brazil), were allocated in a conventional poultry house, weighed and placed in individual wire cages (0.33 m length × 0.46 m deep × 0.40 m height) until the end of the experiment at 70 weeks of age. Hens had free access to water and mash feeds. The lighting program was a 16L:8D cycle.

Hens were distributed by weight and egg production before starting the experiment in a completely randomized block design, where each treatment was composed of 8 White Plymouth Rock and 8 Red Rhodes Island breeder hens. Hens were subjected to a 4-week adjustment period to the experimental diets and then fed the experimental feeds from 55 to 70 weeks of age. Birds were fed 4 dietary treatments with 16 replicates, and the experimental unit was the individually caged hen. Measurements were done in 28 d periods, defined as 55 to 58, 59 to 62, 63 to 66 and 67 to 70 weeks of age.

### 2.2. Experimental Diets

All ingredients utilized during the study were from the same batch. A common control diet (Table 1), formulated of corn, soybean meal and wheat bran, was mixed, and afterwards the additives were supplemented to obtain the other dietary treatments. Treatments consisted of control (control; without additives); control + probiotic (control diet supplemented with *Bacillus subtilis* PB6 probiotic); control + CrProp (control supplemented with chromium propionate); and control + probiotic + CrProp (supplemented with both the probiotic and chromium propionate) diets.

The probiotic was supplemented at 500 g/ton from a commercial *Bacillus subtillis* PB6, PTA-6737 + 98% calcium carbonate (CLOSTAT^®^ Dry; Kemin Industries Inc., Des Moines, IA, USA) with 2.0 × 10^11^ CFU/g. The organic chromium was supplemented at 50 g/ton from a commercial product (KemTRACE^®^ Chromium 0.4% Dry; Kemin Industries Inc., Des Moines, IA, USA), an organic-compliant form of chromium propionate.

### 2.3. Hen Performance and Egg Quality

Hens were fed twice a day and eggs were collected four times per day. Egg production by each hen was recorded daily. Feed intake (g/bird/day), feed conversion (kg feed/kg eggs and kg feed/dozen eggs), egg production (%), egg loss (%) and dirty eggs (%) were grouped per period of 28 d. The egg loss (%) was considered as eggs that were broken, cracked, porous or thin-shelled.

Daily egg mass was calculated by multiplying the laying rate (%) by the average weight of eggs (g) divided by 100. At the end of each 28 d period, average egg weight, specific weight, albumen height, percentage and shell thickness were measured for 4 consecutive days. All intact eggs from each hen were identified and individually weighed. The specific weight was determined using the flotation method in saline solution, where seven saline solutions were prepared, ranging from 1.070 to 1.094 g/cm^3^ with a variation of 0.004 g/cm^3^ for each solution. After the test, all eggs were used to determine the albumen height. Measurements in millimeters (mm) were related to egg weight to determine the Haugh unit: HU log 100(H + 7.57 − 1.7 W^0.37^), in which H = albumen height (mm) and W = egg weight (g).

Yolk and albumen weights were also obtained to determine yolk and albumen percentages of the entire egg. The yolk index (YI) was assessed by measuring yolk width (YW) and yolk height (YH), and was the ratio between these two parameters as YI = YH/YW. The percentage of moisture and total solids of yolks were determined using one egg per cage (method 950.46 [22]). The yolk samples were placed in individual plastic drying containers and taken to a forced-air oven with a temperature of 55 °C for 72 h (Marconi, MA035, Piracicaba, SP, Brazil). Then, total solids were evaluated in an oven at 105 °C for 24 h (method 934.01 [23]).

Shells were washed and dried at room temperature for 72 h, then weighed using a precision digital scale (0.001 g; Bioscale, São Paulo, SP, Brazil). Shell percentage was expressed relative to the egg weight. After weighing the shells, shell thickness was measured at 3 points in the central region of each shell without the external membrane using a digital micrometer (Mitutoyo Sul Americana, Sao Paulo, SP, Brazil). 

All intact eggs produced on day 28 of each period were used for the measurement of shell strength (N) and yolk strength (N), which were obtained with a texturometer, a TA.XT2 Texture Analyzer with a cylindrical, stainless steel 6 mm probe (Texture Technologies Corp. and Stable Micro Systems Ltd., Hamilton, MA, USA) following the methodology described by Stefanello et al. [24]. After shell strength evaluation, the egg was broken to measure the yolk vitelline membrane strength, which was also obtained with a texturometer, a TA.XT2 Texture Analyzer with a cylindrical, stainless steel 2 mm probe.

### 2.4. Blood Chemistry

Blood was collected from 10 random hens before the start of the experimental period. At the end of each 28 d period, blood was collected from the wing vein of 8 hens per treatment and centrifuged (3400 RPM for 20 min), and the obtained serum was stored at −80 °C. The serum was used to determine glucose, total cholesterol, albumin and triglycerides using a BS-120 automatic biochemical analyzer (Mindray Headquarters, Nanshan, Shenzhen, China). Commercial kits (Bioclin^®^, Quibasa, Belo Horizonte, MG, Brazil) were used following the methodology proposed by the manufacturer for albumin, cholesterol, triglycerides and glucose. Serum cortisol (*n* = 32 per period) was determined using a chemiluminescence immunoassay kit. Samples were automatically diluted by the biochemical analyzer in the proportion of 1:10 (sample to distilled water), reducing the effects of lipemia, hemolysis and jaundice that any sample could present.

### 2.5. Excreta Moisture

At the end of each 28 d period, excreta were collected on trays covered with plastic placed under the cages. Excreta samples from each experimental unit were pooled by cage, mixed and stored at −20 °C until analysis. Subsequently, excreta were weighed and dried in a forced-air oven at 55 °C until reaching constant weight and ground to pass a 0.5 mm screen (Tecnal, R-TE-648, Sao Paulo, SP, Brazil). Moisture analysis of excreta was performed after oven-drying the samples at 105 °C for 16 h (method 934.01 [23]).

### 2.6. Total Tract Metabolizability

On the last day of the experiment (at 70 weeks of age), excreta were collected per cage to evaluate the apparent metabolizable energy (AME) and the apparent total tract metabolizability coefficients of dry matter (DM), nitrogen and energy. Celite at 1% was used in each experimental feed, and diets with markers were provided for 3 days before the excreta collection. 

Excreta were collected twice daily for 2 days, immediately mixed and pooled by cage and stored at −20 °C until analysis. Prior to calorimetry, excreta were dried in a forced-air oven at 55 °C and ground to pass a 0.5 mm screen. Analysis of the concentration of dry matter of diets and excreta was performed after oven-drying the samples at 105 °C for 16 h. Diets and excreta were also analyzed for gross energy (GE) using an adiabatic bomb calorimeter (Parr Instrument Company, 6400 Calorimeter, Moline, IL, USA) with benzoic acid as a calibration standard. The nitrogen concentration of diets and excreta was determined via the dry combustion method using a CN analyzer (Thermo-Finnigan Flash EA 1112, Waltham, MA, USA). Acid insoluble ash concentrations in the diet and excreta samples were determined using the method described by Vogtmann et al. [25] and Choct and Annison [26]. 

Apparent total tract metabolizability coefficients of DM, nitrogen and energy were calculated using the following equation [27,28]:

Metabolizability coefficient = 1 − [(Mi/Mo) × (Eo/Ei)],

where Mi and Mo are the concentration of acid insoluble ash in diet and excreta (g/kg of DM) samples, respectively; Ei and Eo are the concentration of nitrogen or DM (g/kg of DM) or GE (kcal/kg of DM) in diet and excreta samples, respectively. 

The AME (kcal/kg) of experimental feeds was calculated using the analyzed content of acid insoluble ash and GE as previously described by Kong and Adeola [27], Stefanello et al. [28] and Haetinger et al. [29].

### 2.7. Statistical Analysis

Data were submitted to the normality test and Levene’s test for homogeneity of variance and were transformed using the arcsine square root percentage (z = arcsine (sqrt (y/100)) whenever not normally distributed [30]. Data were subjected to one-way analysis of variance using the MIXED procedure of SAS [31], and means were compared by the Tukey test at *p* < 0.05.

## 3. Results

Analysis of *Bacillus subtilis* PB6 in the experimental diets showed that the supplemental probiotic had in-feed concentration in agreement with the expected values. *Bacillus subtilis* PB6 was not detected in the control or control + CrProp feeds; however, the recovery of *Bacillus subtilis* in the control + probiotic feed was 6.8 × 10^7^ CFU/g and the control + probiotic + CrProp feed had 7.0 × 10^7^ CFU/g. Chromium was supplemented at 0.20 mg/kg of feed and it was not detected in water samples collected on the first and last day of the study.

No effect was observed between the two strains of layer breeders, previously blocked in the experimental design, and only treatments were considered as an independent variable. There were no effects (*p* > 0.05) of dietary treatments on body weight, feed intake, egg loss, dirty eggs and mortality evaluated per 28 d period or in the overall period.

Productive performance and excreta moisture of layer breeders are presented in Table 2 and demonstrate that the probiotic and organic chromium supplementation improved FCR, egg production and egg mass (*p* < 0.05). From 59 to 62 and 63 to 66 weeks of age, hens fed the control + probiotic + CrProp diet had lower excreta moisture compared to birds fed the control diet (*p* = 0.047 and *p* = 0.052, respectively); however, the excreta moisture was not different among treatments in the overall period from 55 to 70 weeks of age. 

Egg production increased when layer breeders were fed the control + CrProp or control + probiotic + CrProp diets from 63 to 66 weeks of age (*p* = 0.042), and hens fed control + probiotic or control + probiotic + CrProp diets presented higher egg mass (*p* = 0.059) and lower FCR (kg/kg) (*p* = 0.025) in this period. From 67 to 70 weeks of age, layer breeders fed diets supplemented with the probiotic, CrProp or both had higher egg production and egg mass than the non-supplemented hens (*p* < 0.05). The combination between the probiotic and chromium supplemented in hens’ diets resulted in the lowest FCR in kg/kg and kg/dz from 67 to 70 weeks of age. 

In the overall period, egg production increased when hens were fed the control + probiotic or control + probiotic + CrProp diets compared to the control (*p* = 0.043). Additionally, layer breeders fed diets supplemented with probiotic, CrProp or both additives had higher egg mass and lower FCR (kg/kg) than the non-supplemented hens (*p* < 0.05). 

Egg quality and eggshell quality results are presented in Table 3. There were no effects (*p* > 0.05) of dietary treatments on the Haugh unit, albumen and yolk percentage, yolk strength, yolk index and total solids evaluated per 28 d period or in the overall period. However, shell thickness increased when hens were fed diets supplemented with the probiotic, CrProp or both additives compared to the control in all periods as well as from 55 to 70 weeks of age (*p* < 0.05). Shell strength also increased in hens fed the control + CrProp and control + probiotic + CrProp diets compared to the control and control + probiotic diets (*p* < 0.05) in all 28 d periods as well as in the overall period. From 63 to 66, 67 to 70 and 55 to 70 weeks of age, diets supplemented with the additives individually or in combination resulted in higher (*p* < 0.05) specific weight and shell percentage compared to the non-supplemented control diet. 

The results of serum albumin, cholesterol, glucose and triglycerides at 50 weeks of age (*n* = 10), before the start of the experimental period, were 2.70 g/dL, 176 mg/dL, 210 mg/dL and 2012 mg/dL, respectively. Serum cortisol (*n* = 32 per period) was determined each 28 d period and there were no differences (*p* > 0.05) between cortisol concentrations for all treatments and periods, and the obtained mean values were 0.05 mcg/dL or lower. In the experimental period, dietary treatments did not affect (*p* > 0.05) albumin and triglycerides (Table 4); however, in the overall period, cholesterol and glucose serum concentration decreased (*p* = 0.009 and *p* = 0.037, respectively) when hens were fed the control + probiotic or control + probiotic + CrProp diets compared to the control diet.

The total tract metabolizability and AME of layer breeders at 70 weeks of age are shown in Table 5. The AME was not significantly different among treatments; however, metabolizability coefficients of dry matter and energy increased when hens were fed diets supplemented with the probiotic or the probiotic + chromium propionate compared to the control diet (*p* < 0.05). Hens fed diets supplemented with the probiotic + CrProp had the highest total tract metabolizability of nitrogen (*p* = 0.001).

## 4. Discussion

Layer breeders fed diets supplemented with additives can provide useful results for commercial laying hens and broiler breeder hens. The present study had the objective of evaluating the effects that *Bacillus subtilis* PB6 and chromium propionate products would have on serum biochemical parameters, excreta moisture, energy utilization, egg quality and productive performance of late-phase laying breeder hens. 

Although probiotics and chromium present different main functions when supplemented in poultry feeds, these additives have some similar actions on stress control, improving the health status and immune system of poultry, which may result in enhanced bird performance. Marked benefits in performance and egg quality were observed when hens’ diets were supplemented with both additives. Additionally, there are different genera of microorganisms commonly used as probiotics as well as variable sources of chromium, which may influence the obtained results when added in poultry feeds [32,33]. Therefore, *Bacillus subtilis* and organic chromium should be more explored for layers and breeder hens, because more data are needed considering the importance of these productions in the poultry industry. 

The use *of Bacillus subtilis* PB6 (ATCC-PTA 6737) has been applied in the broiler feed industry, being well-documented in the literature [5,8]. For broilers, in addition to improving intestinal microflora, beneficial effects such as stimulated digestion, enhanced performance [8,34] and reduced mortality caused by disease [35] have been reported. There are also published data available on the effect of *Bacillus subtilis* on hen performance and egg quality [7,10,36]. However, little information is available on the role of *Bacillus subtilis* PB6 in layers’ or breeders’ production. 

In the present study, *Bacillus subtilis* PB6 supplementation improved layer breeders’ performance, egg and eggshell quality. These results corroborated the study conducted by Darsi and Zhaghari [11], where 63-week-old broiler breeder hens fed diets supplemented with *Bacillus subtilis* PB6 (2 × 10^7^ CFU/g) presented higher egg production compared to hens fed non-supplemented diets. These authors observed similar egg weight, but increased Haugh unit and shell thickness from 60 to 63 weeks of age.

Darsi and Zhaghari [11] also observed that *Bacillus subtilis* PB6 supplemented in diets for broiler breeders from 56 to 63 weeks of age resulted in a lower percentage of cracked and dirty eggs, obtaining a positive effect on settable eggs and hatchling healthiness. Additionally, other studies reported decreased broken, cracked and shell-less eggs when probiotics were used [4,32,37]. In the present study, *Bacillus subtilis* PB6 supplementation decreased dirty and cracked eggs by 3% compared to non-supplemented hens in the overall period; however, the small number of layer breeders available for this research did not permit detection of small, statistically significant differences. The same reason explains the absence of hatchability results. 

The increased eggshell thickness was already associated with the reduction in the number of cracked eggs, and it was suggested that the decreased damaged eggs could be caused by the increased calcium retention in layers [11,32]. In the current study, layer breeders fed diets supplemented with *Bacillus subtilis* PB6 probiotic presented higher specific weight and shell thickness as well as shell strength and shell percentage, mainly from 67 to 70 weeks of age or in the overall period. 

In the study conducted by Sobczak and Kozłowski [10], increased percentage, thickness and strength of eggshells were obtained without modifying serum calcium, phosphorus, triglycerides and cholesterol in 42-week-old Lohmann Brown laying hens fed diets supplemented with *Bacillus subtilis* PB6 (1 × 10^8^ CFU/kg feed) compared to the non-supplemented group. Fathi et al. [37] evaluated three different breeds of layers from 36 to 48 weeks of age fed 4 × 10^9^ CFU/g of *Bacillus subtilis*, and reported increased eggshell percentage, thickness and strength with lower cholesterol compared to non-supplemented diets. 

Some studies have shown that probiotic supplementation increased levels of calcium and decreased cholesterol levels in the serum of Brown laying hens [4,38,39]. Gilliland et al. [40] suggested that certain microorganisms present in probiotics might assimilate cholesterol from the gastrointestinal tract for their metabolism, thus reducing the amount of absorbed cholesterol. Probiotic bacterial strains are also able to inhibit the activity of hydroxymethyl-glutaryl-coenzyme A in the gastrointestinal tract [41] or modify the enterohepatic cycle and reduce cholesterol through assimilating dietary cholesterol into bacterial cells [42].

Data on the digestibility and metabolizability of energy and nutrients using *Bacillus subtilis* PB6 supplemented in hens’ feeds are scarce. In the present study, laying breeder hens fed diets supplemented with this probiotic from 55 to 70 weeks of age had improved egg production and eggshell quality, which were supported by increased energy, nitrogen and dry matter metabolizability. In broiler diets, probiotic supplementation has been related to improved performance along with increased AME and ileal digestibility of nutrients [43], because this additive may be able to enhance the maintenance and barrier function of the intestinal epithelium [1]. Dietary supplementation of *Bacillus subtilis* also allowed for a reduction in excreta moisture, thereby preventing problems caused by wet litter in poultry farms [7]. Excreta moisture was reduced in the current study, which was similar to findings by Ribeiro Jr. et al. [36], where lower excreta moisture was reported when 25- to 45-week-old layers were fed a *Bacillus subtilis* at 3 or 8 × 10^5^ CFU/kg diet. 

To further substantiate the use of probiotic and chromium as potential, more beneficial effects were obtained when hens were fed *Bacillus subtilis* PB6 + CrProp in the last period of age, where environmental temperatures were hotter. In the present study, a conventional poultry house was used, representing the climate and environmental conditions where hens have been raised in South and Southeastern regions of America, which is characterized by hot summers and mild winter temperatures. In these regions, hen producers do not prioritize investing in the environment of their farms. Additionally, the comparison of temperatures was not an objective of this study, and for this reason, it was not presented. Nevertheless, averaged maximum and minimum temperatures monitored on a daily basis inside the shed were 21.6 °C and 12.4 °C, 25.5 °C and 13.0 °C, 28.8 °C and 18.0 °C and 30.9 °C and 19.1 °C, respectively, from 55 to 58, 59 to 62, 63 to 66 and 67 to 70 weeks of age. The mean air humidity was 66.0%.

In the current study, CrProp supplementation improved the egg production, FCR (kg/kg) and egg mass of laying breeder hens. Ma et al. [21] supplemented increasing levels of CrProp in layers diets and observed enhanced egg production without affecting egg weight and FCR in late-phase brown laying hens. Egg production and FCR were also improved when CrProp was supplemented in diets for laying ducks under heat-stressed conditions without modifying FI, as reported by Chen et al. [16]. These authors also indicated that CrProp was probably effective in alleviating the negative effects of heat stress. Marked effects of CrProp were observed by the increased shell strength as well as shell thickness and percentage, with improved specific weight. Additionally, the majority of eggshell quality effects were observed after 67 weeks of age in the present study. Improved shell thickness was also reported by Ma et al. [21] in laying hens fed a 0 to 0.60 mg Cr/kg diet from 60 to 68 weeks of age. 

Chromium supplementation was previously reported to decrease blood total cholesterol, low-density lipoprotein cholesterol and triglyceride [44,45]. In the present study, cholesterol and glucose decreased when hens were fed diets supplemented with CrProp from 55 to 70 weeks of age. However, albumin, triglycerides and cortisol were not affected. Bahrami et al. [46] observed reduced cortisol levels in the serum of broilers under heat stress conditions that were fed diets supplemented with high Cr supplementation (0, 0.80 or 1.20 mg). Since stress might have multiple origins, and cortisol could be a tool to evaluate stress tolerance, studies reported the association between chromium and stress metabolism through decreased sensitivity to stress and reduced concentration of cortisol in blood [47]. 

As mentioned above, along with stressful conditions and bird species, the results of probiotic supplementation in bird diets can be variable depending on factors related to microbial strains and supplemental doses. In the same context, contradictory results on performance, egg quality and blood biochemistry concentration can be expected due to chromium forms, sources and levels, as well as laying bird species, ages or production systems. The combination of *Bacillus subtilis* PB6 and chromium propionate seemed to beneficially affect the performance and eggshell quality of late-phase laying breeder hens.

## 5. Conclusions

In conclusion, diets supplemented with *Bacillus subtilis* PB6, chromium propionate or a combination of the two resulted in improved egg production, feed conversion ratio and eggshell quality as well as nutrient metabolizability, without modifying the main serum biochemical parameters in laying breeder hens from 55 to 70 weeks of age. Highlighted improvements in hen performance and eggshell quality were observed when hens were fed diets supplemented with a combination of probiotic *Bacillus subtilis* PB6 + chromium propionate. This study expands our understanding concerning combined probiotic and organic chromium supplementation for laying breeder hens.

## Figures and Tables

**Table 1:** Ingredient and nutrient composition of the control diet.

Item	Control Diet (55 to 70 Weeks of Age)
Ingredients, %	
Corn	58.52
Soybean meal, 46 % CP	19.64
Wheat bran	4.00
Soybean oil	2.36
Dicalcium phosphate	1.52
Limestone	12.84
Salt	0.43
DL-Methionine, 99%	0.30
L-Lysine-HCl, 78%	0.15
L-Threonine, 98.5%	0.09
Mineral and vitamin premix ^1^	0.15
Nutrient and energy composition, % or as shown	
AME, kcal/kg	2750
Crude protein	14.50
Calcium	4.30
Available phosphorus	0.35
Total phosphorus	0.53
Sodium	0.18
Potassium	0.59
Chloride	0.35
Dig. Lys ^2^	0.75
Dig. Met + Cys	0.70
Dig. Thr	0.58
Dig. Trp	0.15

^1^ Composition per kilogram of feed: vitamin A, 8000 IU; vitamin D3, 2000 IU; vitamin E, 30 IU; vitamin K3, 2 mg; thiamine, 2 mg; riboflavin, 6 mg; pyridoxine, 2.5 mg; cyanocobalamin, 0.012 mg, pantothenic acid, 15 mg; niacin, 35 mg; folic acid, 1 mg; biotin, 0.08 mg; iron, 40 mg; zinc, 80 mg; manganese, 80 mg; copper, 10 mg; iodine, 0.7 mg; and selenium, 0.3 mg. ^2^ Digestible-amino-acids-to-digestible-Lys ratios were maintained at TSAA 0.94; Thr 0.77; Val 0.77; Trp 0.20; Arg 1.13; and Ile 0.67.

**Table 2:** Productive performance and excreta moisture of layer breeders fed diets supplemented with the probiotic and chromium propionate from 55 to 70 weeks of age.

Item	BW, g	EP ^1^, %	FI ^2^, g/hen/d	Egg Loss, %	Dirty Eggs, %	Egg Mass, g	FCR ^3^, kg/kg	FCR, kg/dz	Excreta Moisture, %
Period of 55 to 58 weeks of age									
Control	2163	69.9	130	11.2	6.7	40.3	3.25	2.22	79.9
Control + probiotic ^4^	2161	72.6	130	9.1	6.4	43.3	3.03	2.20	79.6
Control + CrProp ^5^	2095	71.9	128	9.4	6.4	41.6	3.09	2.15	79.2
Control + probiotic + CrProp	2112	73.2	129	3.9	6.4	42.4	3.04	2.07	79.0
SEM	22.8	0.88	1.7	1.6	0.9	0.52	0.05	0.04	0.19
*p*-value	0.648	0.574	0.918	0.394	0.974	0.216	0.464	0.620	0.355
Period of 59 to 62 weeks of age									
Control	2144	67.8	131	6.7	8.5	39.1	3.43	2.49	79.4 ^a^
Control + probiotic	2159	72.6	129	3.7	5.3	42.9	3.07	2.19	77.8 ^b^
Control + CrProp	2062	70.7	127	4.5	7.2	41.9	3.05	2.10	79.0 ^ab^
Control + probiotic + CrProp	2076	72.8	130	2.8	3.7	42.4	3.09	2.08	77.5 ^b^
SEM	23.0	1.19	1.6	2.0	1.3	0.70	0.06	0.06	0.29
*p*-value	0.345	0.429	0.858	0.517	0.621	0.237	0.102	0.068	0.047
Period of 63 to 66 weeks of age									
Control	2143	64.6 ^b^	129	9.0	9.2	37.6 ^b^	3.49 ^a^	2.41	79.0 ^a^
Control + probiotic	2177	68.9 ^ab^	127	6.2	6.1	41.0 ^a^	3.13 ^b^	2.25	77.3 ^ab^
Control + CrProp	2061	70.1 ^a^	127	8.1	7.8	40.2 ^ab^	3.19 ^ab^	2.21	77.3 ^ab^
Control + probiotic + CrProp	2078	72.3 ^a^	123	2.8	7.1	41.9 ^a^	2.94 ^b^	2.17	76.2 ^b^
SEM	24.7	0.99	1.90	1.4	1.3	0.61	0.07	0.05	0.40
*p*-value	0.301	0.042	0.718	0.392	0.862	0.059	0.025	0.341	0.052
Period of 67 to 70 weeks of age									
Control	2099	63.4 ^b^	121	8.3	10.8	36.8 ^b^	3.39 ^a^	2.53 ^a^	77.4
Control + probiotic	2170	69.2 ^a^	123	6.2	7.3	41.0 ^a^	3.05 ^ab^	2.19 ^ab^	76.9
Control + CrProp	2008	69.5 ^a^	120	5.9	5.8	41.4 ^a^	2.89 ^b^	2.18 ^ab^	76.2
Control + probiotic + CrProp	2059	70.8 ^a^	122	5.9	5.5	42.4 ^a^	2.95 ^b^	2.10 ^b^	75.5
SEM	25.8	1.04	2.13	1.1	1.5	0.65	0.07	0.08	0.43
*p*-value	0.150	0.049	0.933	0.859	0.573	0.009	0.059	0.190	0.422
Overall period (55 to 70 weeks of age)									
Control	2138	66.4 ^b^	128	8.8	8.8	38.4 ^b^	3.39 ^a^	2.41 ^a^	78.9
Control + probiotic	2167	70.8 ^a^	127	6.3	6.3	42.1 ^a^	3.07 ^b^	2.21 ^ab^	78.0
Control + CrProp	2056	70.5 ^ab^	125	6.5	7.0	41.3 ^a^	3.06 ^b^	2.16 ^b^	77.9
Control + probiotic + CrProp	2081	72.3 ^a^	126	5.7	3.9	42.3 ^a^	3.00 ^b^	2.10 ^b^	77.0
SEM	22.3	0.77	1.42	0.99	0.86	0.46	0.05	0.04	0.26
*p*-value	0.279	0.043	0.914	0.715	0.230	0.009	0.012	0.051	0.443

^1^ EP = egg production. ^2^ FI = feed intake. ^3^ FCR = feed conversion ratio. ^4^ *Bacillus subtilis* PB6, PTA-6737 supplemented at 500 g/ton. ^5^ Chromium propionate supplemented at 50 g/ton. ^a,b^ Means with different superscript letters differ significantly (*p* < 0.05) based on the Tukey test.

**Table 3:** Egg and eggshell quality of layer breeders fed diets supplemented with the probiotic and chromium propionate from 55 to 70 weeks of age.

Item	Egg Weight, g	Specific Weight, g/cm^3^	Haugh Unit	Albumen, %	Yolk, %	Yolk Strength, N	Yolk Index	Total Solids, %	Shell, %	Thickness, mm	Shell Strength, N
Period of 55 to 58 weeks of age											
Control	57.1	1084	89.1	62.2	28.8	7.9	0.47	56.3	8.5	0.352 ^b^	26.3 ^b^
Control + probiotic ^1^	59.7	1086	89.6	62.6	29.0	7.9	0.49	55.8	8.9	0.372 ^a^	26.4 ^b^
Control + CrProp ^2^	58.0	1085	90.4	64.5	28.3	7.8	0.49	55.7	8.8	0.373 ^a^	29.6 ^a^
Control + probiotic + CrProp	58.2	1084	89.7	62.6	28.5	7.8	0.49	55.9	9.0	0.376 ^a^	29.7 ^a^
SEM	0.49	0.56	0.57	0.47	0.27	0.02	0.003	0.23	0.09	0.003	0.24
*p*-value	0.294	0.419	0.885	0.330	0.828	0.428	0.479	0.781	0.238	0.016	0.001
Period of 59 to 62 weeks of age											
Control	57.8	1079 ^b^	88.6	63.4	29.0	7.5	0.47	54.2	8.1	0.343 ^b^	25.1 ^b^
Control + probiotic	59.1	1084 ^a^	89.0	63.4	29.1	7.6	0.48	54.0	8.5	0.366 ^a^	25.3 ^b^
Control + CrProp	57.9	1084 ^a^	90.8	63.2	28.6	7.5	0.49	54.9	8.7	0.365 ^a^	28.7 ^a^
Control + probiotic + CrProp	59.0	1083 ^a^	90.4	63.6	29.1	7.5	0.48	54.5	8.7	0.360 ^a^	28.7 ^a^
SEM	0.49	0.65	0.71	0.41	0.22	0.01	0.003	0.23	0.12	0.003	0.26
*p*-value	0.695	0.014	0.648	0.987	0.807	0.748	0.136	0.533	0.172	0.050	0.001
Period of 63 to 66 weeks of age											
Control	58.2	1079 ^b^	88.9	61.6	29.9	7.4	0.44	53.7	8.1 ^b^	0.347 ^b^	24.1 ^b^
Control + probiotic	59.6	1082 ^ab^	89.3	61.8	29.4	7.4	0.45	54.5	8.7 ^a^	0.364 ^ab^	24.2 ^b^
Control + CrProp	57.6	1084 ^a^	91.0	62.6	28.9	7.4	0.46	52.7	8.9 ^a^	0.365 ^a^	28.0 ^a^
Control + probiotic + CrProp	59.0	1081 ^ab^	89.4	62.6	29.2	7.5	0.45	53.2	8.6 ^a^	0.367 ^a^	28.1 ^a^
SEM	0.51	0.55	0.59	0.25	0.21	0.01	0.003	0.42	0.09	0.004	0.28
*p*-value	0.535	0.020	0.599	0.293	0.425	0.849	0.209	0.487	0.010	0.055	0.001
Period of 67 to 70 weeks of age											
Control	58.1	1079 ^b^	88.1	62.4	29.9	7.5	0.46	53.1	8.2 ^b^	0.345 ^b^	24.6 ^b^
Control + probiotic	60.5	1084 ^a^	88.0	62.1	29.4	7.5	0.47	53.3	8.8 ^a^	0.366 ^a^	24.9 ^b^
Control + CrProp	58.3	1083 ^a^	90.2	63.4	28.9	7.6	0.47	53.2	8.7 ^a^	0.369 ^a^	28.6 ^a^
Control + probiotic + CrProp	60.1	1083 ^a^	88.6	62.8	29.2	7.5	0.46	53.5	8.7 ^a^	0.370 ^a^	28.6 ^a^
SEM	0.49	0.53	0.57	0.26	0.20	0.01	0.003	0.14	0.09	0.003	0.29
*p*-value	0.198	0.008	0.488	0.350	0.321	0.112	0.545	0.713	0.045	0.003	0.001
Overall period (55 to 70 weeks of age)											
Control	57.8	1080 ^b^	88.7	62.4	29.4	7.6	0.46	54.3	8.2 ^b^	0.347 ^b^	25.1 ^c^
Control + probiotic	59.7	1084 ^a^	88.9	62.5	29.2	7.6	0.47	54.4	8.7 ^a^	0.367 ^a^	25.2 ^b^
Control + CrProp	57.9	1084 ^a^	90.6	63.4	28.7	7.6	0.48	54.1	8.8 ^a^	0.368 ^a^	28.7 ^a^
Control + probiotic + CrProp	59.1	1083 ^a^	89.5	63.0	29.0	7.6	0.47	54.3	8.8 ^a^	0.370 ^a^	28.8 ^a^
SEM	0.45	0.49	0.51	0.26	0.17	0.01	0.003	0.14	0.08	0.003	0.26
*p*-value	0.369	0.014	0.555	0.446	0.499	0.160	0.186	0.892	0.028	0.003	0.001

^1^ *Bacillus subtilis* PB6, PTA-6737 supplemented at 500 g/ton. ^2^ Chromium propionate supplemented at 50 g/ton. ^a–c^ Means with different superscript letters differ significantly (*p* < 0.05) based on the Tukey test.

**Table 4:** Blood biochemical results of layer breeders fed diets supplemented with the probiotic and chromium propionate from 55 to 70 weeks of age.

Item^1^	Albumin, g/dL	Cholesterol, mg/dL	Glucose, mg/dL	Triglycerides, mg/dL
Period of 55 to 58 weeks of age				
Control	2.14	149	202	1887
Control + probiotic ^2^	2.18	149	193	1774
Control + CrProp ^3^	2.31	148	194	1902
Control + probiotic + CrProp	2.10	129	190	1498
SEM	0.04	6.63	2.33	132.9
*p*-value	0.211	0.622	0.324	0.704
Period of 59 to 62 weeks of age				
Control	2.13	157 ^a^	201 ^a^	1917
Control + probiotic	2.16	157 ^a^	186 ^b^	1978
Control + CrProp	2.20	132 ^ab^	181 ^b^	1551
Control + probiotic + CrProp	2.08	119 ^b^	188 ^ab^	1391
SEM	0.03	5.44	2.54	110.1
*p*-value	0.386	0.020	0.034	0.172
Period of 63 to 66 weeks of age				
Control	2.44	128	179	1626
Control + probiotic	2.30	115	173	1308
Control + CrProp	2.39	123	170	1404
Control + probiotic + CrProp	2.30	112	181	1251
SEM	0.03	5.55	1.71	96.9
*p*-value	0.367	0.143	0.080	0.556
Period of 67 to 70 weeks of age				
Control	2.55	147	185	1648
Control + probiotic	2.59	134	186	1591
Control + CrProp	2.50	118	183	1196
Control + probiotic + CrProp	2.51	116	179	1478
SEM	0.04	5.44	2.33	101.2
*p*-value	0.900	0.157	0.760	0.414
Overall period (55 to 70 weeks of age)				
Control	2.33	149 ^a^	192 ^a^	1770
Control + probiotic	2.33	139 ^ab^	185 ^ab^	1663
Control + CrProp	2.36	129 ^bc^	182 ^b^	1514
Control + probiotic + CrProp	2.25	119 ^c^	183 ^b^	1404
SEM	0.02	3.84	1.32	67.3
*p*-value	0.231	0.009	0.037	0.235

^1^ Serum cortisol was determined each 28 d period and the obtained means were 0.05 mcg/dL or lower than the limit of detection for all experimental diets and periods (*p* > 0.05). The results of serum albumin, cholesterol, glucose and triglycerides at 50 weeks of age (*n* = 10) before the start of the experimental period were 2.70 g/dL, 176 mg/dL, 210 mg/dL and 2012 mg/dL, respectively. ^2^ *Bacillus subtilis* PB6, PTA-6737 supplemented at 500 g/ton. ^3^ Chromium propionate supplemented at 50 g/ton. ^a–c^ Means with different superscript letters differ significantly (*p* < 0.05) based on the Tukey test.

**Table 5:** Coefficient of total tract metabolizability and apparent metabolizable energy (AME) of layer breeders fed diets supplemented with the probiotic and chromium propionate at 70 weeks of age.

Item	Dry Matter	Nitrogen	Energy	AME, kcal/kg
Control	0.67 ^b^	0.56 ^c^	0.80 ^b^	3155
Control + probiotic ^1^	0.72 ^a^	0.61 ^b^	0.84 ^a^	3215
Control + CrProp ^2^	0.71 ^a^	0.65 ^ab^	0.82 ^b^	3210
Control + probiotic + CrProp	0.72 ^a^	0.68 ^a^	0.85 ^a^	3226
SEM	0.005	0.010	0.004	16.61
*p*-value	0.004	0.001	0.001	0.249

^1^ *Bacillus subtilis* PB6, PTA-6737 supplemented at 500 g/ton. ^2^ Chromium propionate supplemented at 50 g/ton. ^a–c^ Means with different superscript letters differ (*p* < 0.05) based on Tukey test.

## Data Availability

Data presented in this study are available on request from the corresponding author.
